# Catalyst Accessibility and Acidity in the Hydrocracking of HDPE: A Comparative Study of H-USY, H-ZSM-5, and MCM-41 Modified with Ga and Al

**DOI:** 10.3390/molecules29174248

**Published:** 2024-09-07

**Authors:** Cátia S. Costa, M. Rosário Ribeiro, João M. Silva

**Affiliations:** 1 Centro de Química Estrutural, Institute of Molecular Sciences, Instituto Superior Técnico, Universidade de Lisboa, 1049-001 Lisboa, Portugal; catia.s.costa@tecnico.ulisboa.pt (C.S.C.); jmsilva@deq.isel.ipl.pt (J.M.S.); 2 Chemical Engineering Department, Instituto Superior de Engenharia de Lisboa, Instituto Politécnico de Lisboa, 1959-007 Lisboa, Portugal

**Keywords:** hydrocracking, HDPE, mesoporous silicas, zeolites, acidity, accessibility

## Abstract

Plastic pollution is a critical environmental issue due to the widespread use of plastic materials and their long degradation time. Hydrocracking (HDC) offers a promising solution to manage plastic waste by converting it into valuable products, namely chemicals or fuels. This work aims to investigates the effect of catalyst accessibility and acidity on the HDC reaction of high density polyethylene (HDPE). Therefore, a variety of materials with significant differences in both textural and acidic properties were tested as catalysts. These include H-USY and H-ZSM.5 zeolites with various Si/Al molar ratios (H-USY: Si/Al = 2.9, 15, 30 and 40; H-ZSM-5: Si/Al = 11.5, 40, 500) and mesostructured MCM-41 materials modified with Ga and Al, also with different Si/metal ratios (Si/Al = 16 and 30; Si/Ga = 63 and 82). Thermogravimetric analysis under hydrogen atmosphere was used as a preliminary screening tool to evaluate the potential of the various catalysts for this application in terms of energy requirements. In addition, batch autoclave reactor experiments (T = 300 °C, PH_2_ = 20 bar, t = 60 min) were conducted to obtain further information on conversion, product yields and product distribution for the most promising systems. The results show that the catalytic performance in HDPE hydrocracking is determined by a balance between the acidity of the catalyst and its structural accessibility. Accordingly, for catalyst series where the structural and textural properties do not vary with the Si/Al ratio, there is a clear correlation of the HDPE degradation temperature and of the HDPE conversion with the Si/metal ratio (which relates to the acidic properties). In contrast, for catalyst series where the structural and textural properties vary with the Si/Al ratio, no consistent trend is observed and the catalytic performance is determined by a balance between the acidic and textural properties. The product distribution was also found to be influenced by the physical and chemical properties of the catalyst. Catalysts with strong acidity and smaller pores were observed to favor the formation of lighter hydrocarbons. In addition to the textural and acidic properties of the catalyst, the role of coke formation should not be neglected to ensure a comprehensive analysis of the catalytic performance.

## 1. Introduction

The escalating volume of plastic waste (PW) has raised significant social, economic, and environmental concerns worldwide [1]. In response, the scientific community has made considerable efforts to develop innovative strategies for PW management, seeking alternatives to conventional methods [2,3]. Among these, hydrocracking (HDC) technology has emerged as a promising solution, enabling the conversion of plastic feedstocks into valuable products, the removal of heteroatoms present in PW, the reduction of olefins and aromatics in the final products, and the minimization of coke precursors that cause catalyst deactivation [4,5]. In the HDC reaction, catalysts are critical because they reduce the temperature and time required to achieve high conversions, thereby improving the quality of gas and liquid products [6]. While the typical HDC catalyst is bifunctional [7], i.e., having both an acidic and metallic function, several monofunctional systems have also been effectively used for PW conversion under a reducing H_2_ atmosphere. These include silica, alumina or silica-alumina (SiO_2_ [8], Al_2_O_3_ [9], and SiO_2_/Al2O_3_ [10]), zeolites (H-ZSM-5 [11,12], H-Beta [13,14], HUSY [13,15], H-FER [16], and H-MOR [16,17]), solid super acids (ZrO_2_/SO_4_ [18,19] and Fe_2_O_3_/SO_4_ [20]), and mesoporous silicas (Al-SBA-15 [21] and Al-SBA-16 [21]). Most of these studies focus primarily on how process operating conditions—such as pressure, temperature, and time—affect product distribution, especially liquid products, without fully exploring the fundamental relationship between catalyst properties and cracking efficiency. In addition, research to date has largely focused on energy recovery through fuel production [4,22,23]. However, recent revisions to EU waste legislation state that plastic waste can only be considered recycled if it is reprocessed into new materials that are not intended for use as fuels. As a result, the current focus is shifting from pure energy recovery to the production of valuable chemicals, such as petrochemical feedstocks or monomers. [24]. Therefore, understanding how catalyst acidity and accessibility (i.e., the ease with which a reactant molecule can approach and interact with the internal active sites of a catalyst and with which the products desorb [25]) affect the catalytic performance in the hydrocracking reaction of HDPE as well as the resulting product distribution is essential for designing new and more effective catalytic systems for diverse product applications.

In our previous studies [26,27], thermogravimetric analysis (TGA) was applied to evaluate the potential of various microporous zeolites with large, medium, and small pores (H-USY, H-ZSM-5, H-MOR and H-FER) and similar Si/Al molar ratios (~15) as well as non-acidic mesoporous silicas (SBA-15 and MCM-41) for the HDC of HDPE. The results demonstrated that the high energy consumption associated with the thermal decomposition of HDPE can be greatly reduced by the addition of a microporous acidic zeolite and H-USY and H-ZSM-5 zeolites with large and medium pores, respectively, were found to be the most promising catalysts. Despite the high accessibility of the mesoporous silica, its presence did not lower the degradation temperature of HDPE, most probably due to the lack of acidic character, typical of these materials. The surface acidity of mesoporous silica materials can be enhanced through the incorporation of hetero elements into the parent mesoporous silicas. Generally, aluminum (Al) is used, although boron (B) or some transition metals such as zirconium (Zr), titanium (Ti), iron (Fe), and gallium (Ga) may also be employed [28,29]. The nature of the element introduced, together with its content (Si/metal molar ratio) determine the number, strength, and nature of the acid sites formed, thereby controlling the acid properties of the materials [30].

The objective of this study is to gain further insight into the effect of catalyst accessibility and acidity on the HDC reaction of high-density polyethylene (HDPE). To this end, a variety of materials displaying significant differences in both textural and acidic properties are investigated, including a set of modified mesoporous silica materials and two different zeolite structures identified as the most promising ones from our previous study, one with large pores, H-USY, and the other with medium pores, H-ZSM-5. For each of these types of materials, the respective acidity has been varied by using a wide range of Si/Al molar ratios. A preliminary assessment of the energy requirements is carried out by thermogravimetric analysis (TGA) under a hydrogen atmosphere. Additional experiments are then conducted in a batch autoclave reactor to obtain conversion, product yields, and product distribution data for the most promising systems.

The set of catalytic systems evaluated comprise a medium pore H-ZSM-5 zeolite (Si/Al = 2.9, 15, 30 and 40), a large pore H-USY zeolite (Si/Al = 11.5, 40 and 500), and parent and Al- and Ga-modified MCM-41 (Si/Al = 16 and 30 and Si/Ga = 63 and 82). A comprehensive analysis of the data will be performed to elucidate how variations in catalyst structure and acidity affect the catalytic behavior in the HDC process.

## 2. Results

### 2.1. Catalyst Characterization

The N_2_ sorption isotherms and the pore size distribution (PSD) of pure siliceous MCM-41 (Si-MCM-41), Al- and Ga-modified MCM-41 with different Si/metal ratios, and H-USY and H-ZSM-5 zeolites with different Si/Al molar ratios are presented in Figure S1 of the Supporting Information, while Figure S2 displays the N_2_ sorption isotherms of the H-USY and H-ZSM-5 zeolites with various Si/Al ratios. The resulting textural properties are summarized in Table 1.

Regarding the N_2_ adsorption data, both Al- and Ga-modified mesoporous MCM-41 reveal a type IV isotherm with H1 hysteresis, characteristic of mesoporous solids with a narrow range of uniform mesopores [31], as previously reported for pure siliceous MCM-41 [26,32]. The PSD reveals similar pore diameters for both Al-MCM-41 or Ga-MCM-41 materials and these were analogous to Si-MCM-41. The pore sizes range between 2.9 and 4.4 nm with mean values in the 3.0–3.4 nm range, as determined by the BJH method. Regarding S_BET_ and V_meso_, no significant changes are observed upon the introduction of Al into the MCM-41 structure. However, the incorporation of high Ga contents into Si-MCM-41 leads to a significant reduction in S_BET_. According to the literature [33], when Ga is incorporated into the MCM-41 structure, it tends to replace Si atoms. Since Ga has a larger ionic radius than Si, this substitution can cause distortions in the silica framework, disrupting the uniformity of the mesoporous structure and reducing the surface area. In addition, Ga can lead to the formation of extra-framework Ga species, which may block or reduce the pore size, negatively affecting the mesoporous volume of MCM-41. On the other hand, aluminum, which has a smaller ionic radius than Ga, can replace Si atoms with minimal distortion, thus preserving the textural properties of MCM-41.

The H-USY zeolites with different Si/Al ratios exhibit a combination of type I and type IV isotherms with hysteresis type H3, confirming the presence of a secondary mesoporosity resulting from the dealumination procedure used to obtain the higher Si/Al molar ratios [34,35]. With regard to the other textural properties (S_ext_, V_micro_, V_meso_), it can be seen that an increase in the Si/Al ratio leads to a change in these properties, with an increase in the S_ext_ and V_meso_. Most likely, this is also a result of the secondary mesoporosity generated during the synthesis method.

**Figure 1:**
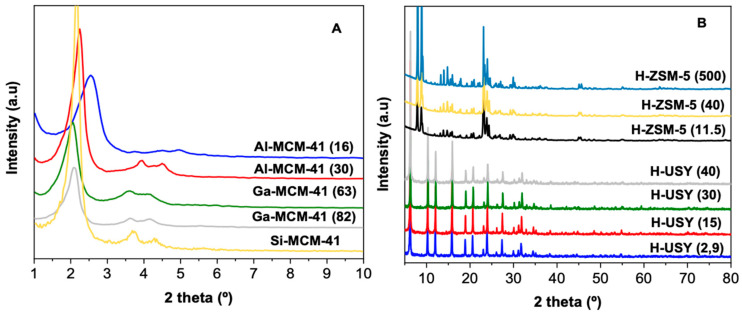
PXRD of Al- and Ga-modified MCM-41 (**A**) and of H-USY and H-ZSM-5 zeolites (**B**) with distinct Si/metal ratios.

Instead, the H-ZSM-5 zeolites with distinct Si/Al reveal a type I isotherm typical of microporous materials. In this case, no significant changes are observed on S_ext_, V_micro_, and V_meso_ by increasing the Si/Al ratio from 11.5 to 40. For H-ZSM-5 (500), however, a subsequent decrease is observed on Sext. As illustrated in Figure 1A, the incorporation of Ga and Al into the MCM-41 structure by direct synthesis results in a broadening of the peaks observed in the powder X-ray diffraction (PXRD) pattern when compared to those of the pure siliceous counterpart. However, the hexagonal structure is clearly identifiable by the three diffraction peaks that, in this symmetry, can be indexed as (100), (110), and (200) [36,37].

Overall, the PXRD data are aligned with the N_2_ adsorption results, as no significant modifications are observed in structural parameters upon Al or Ga incorporation into the pure siliceous MCM-41 structure.

The H-USY zeolites with various Si/Al ratios exhibit PXRD patterns (Figure 1B) characteristic of FAU structure (2θ = 6.2, 10.3, 12.1, 15.9, 18.9, 20.7, 24.0, and 27.5), indicating that the crystalline structure is maintained even after successive dealumination procedures [38]. Furthermore, the absence of significant changes in peak intensity indicates that the crystallinity of the zeolites is not substantially affected by the dealumination process. [39]. The H-ZSM-5 zeolites with different Si/Al ratios also exhibit diffraction peaks characteristic of the MFI structure, (2θ = 7.9, 8.8, 14–17, 23–25) according to the database of zeolitic structures.

The acidic properties of the Al- and the Ga-modified MCM-41 materials and of the H-USY and H-ZSM-5 zeolites were investigated by FTIR-Py. The total amount of Brønsted acid sites (BASs) and Lewis acid sites (LASs) expressed by their respective concentrations at 150°, the amount of strong BASs and LASs expressed by their respective concentrations at 350 °C, and the total acidity are summarized in Table 1.

The incorporation of Al into the parent MCM-41 structure significantly increases the total acidity of the resulting Al-MCM-41 materials and leads to the formation of both LASs and BASs, in a 2:1 ratio. Furthermore, an increase in Al content, i.e., a decrease in the Si/Al ratio, leads to an increase in the total BASs and LASs. In contrast to Al-MCM-41 materials, the incorporation of Ga into the MCM-41 structure results in a slight increase in the total acidity of the catalyst, but its further augmentation does not increase the acidity. In fact, the acidity varies from 0 for pure siliceous MCM-41 to 13 and 31 μmol Py/g for Ga-MCM-41 (63) and Ga-MCM-41 (82), respectively. It should also be noted that all of the centers correspond to LASs.

With respect to the H-USY series, the data show that BASs and LASs, and consequently the total number of available acid sites, decrease in the following order: H-USY (2.9) > H-USY (15) > H-USY (30) > H-USY (40), indicating a relationship between the amount of Al on the zeolite structure and the total acidity of the catalyst, as expected. The same trend is observed for the number of strong BASs, i.e., being able to keep pyridine adsorbed at T = 350 °C. In fact, in the H-USY zeolites, with Si/Al molar ratios of 2.9, 15, 30, and 40, the strong BASs correspond to 69, 45, 36, and 36% of the total BASs, respectively. For the H-ZSM-5 series, an increase in the Si/Al ratio also results in a substantial decrease in BASs, LASs, and the total number of active sites. Again, this is related to the reduction in the zeolite structure of the Al species responsible for the acidic content of the materials [40]. The same trend is observed regarding the number of strong acid sites. Particularly, the ratio between strong and total BASs decreases from 59%, at Si/Al = 11.5, to 19%, at Si/Al = 40.

### 2.2. Preliminary Degradation Experiments

Thermogravimetric analysis (TGA) is a powerful technique for evaluating the thermal and catalytic degradation of polymers. Under catalytic conditions, it is a particularly valuable tool for a preliminary and rapid evaluation of different catalysts, helping to identify the most effective ones in terms of energy requirements [41]. Furthermore, TGA insights can guide the selection and optimization of catalysts for more detailed studies.

A preliminary evaluation of the different sets of materials previously selected, two modified mesoporous silicas and two zeolitic structures, one with large pores and the other with medium pores, was carried out by TGA under a H_2_ atmosphere. For each set of materials, the acidity was varied over a wide range of Si/Al molar ratios. The degradation profiles are shown in Figure S3 of the Supporting Information, while the temperatures at which the mass loss is 5, 50, and 95%, i.e., T_5%_, T_50%_, and T_95%,_ respectively, are given in Table 2. Results for the thermal degradation of HDPE and for the use of the parent mesoporous silica are also shown for comparison purposes.

The data show that the thermal degradation of HDPE occurs at high temperatures (433–488 °C) and that the addition of the parent mesoporous silica (Si-MCM-41) does not alter the degradation profile, as the reaction occurs mainly by the thermal pathway (431–468 °C). The bad performance of Si-MCM-41 is attributed to its lack of acidic character [26]. According to the FTIR-Py data, the incorporation of Al or Ga improves the acidity of Si-MCM-41. However, the nature of the acid centers and the overall acidity is extremely dependent on the incorporated metal (Al or Ga). This leads to very distinct behaviors with respect to HDPE degradation. On the one hand, when the reaction is carried out in the presence of Ga-modified MCM-41, the degradation profiles observed for the thermal process and for both Ga-MCM-41(63) and Ga-MCM-41(82) are very similar (close T_5%_, T_50%_, and T_95%_ values), indicating that no catalytic effect occurs. On the other hand, when the reaction takes place over Al-MCM-41, the HDPE degradation profiles are shifted to lower temperatures, indicating that the incorporation of Al into the MCM-41 structure reduces the energy requirement of the process. Given that both Ga- and Al-MCM-41 catalysts have similar structural and textural properties, it is likely that their different behavior can be attributed to the completely distinct acid properties. The presence of Ga only leads to the formation of Lewis acid sites (LASs), while the incorporation of Al leads to the formation of both BASs and LASs. Furthermore, increasing the number of LASs in the Ga-MCM-41 materials, does not change the degradation temperature profile, whereas increasing the number of BASs in the Al-MCM-41 materials has a beneficial effect. These observations lend support to the hypothesis that a BAS is a prerequisite for HDC reactions [42,43]. In addition, the better performance of the Al- vs. Ga-MCM-41 series can be easily explained by the higher and stronger acidic character of the former materials. Among the Al-MCM-41 materials, the one with a lower Si/Al ratio (16) and a higher number of BASs, shows a reduction of 124 °C at T_5%_ compared to Si-MCM-41, while the one with a higher Si/Al ratio (30) and a much lower number of BAS shows a reduction of only 11°C at T5%. These results demonstrate the beneficial effect of lowering the Si/Al ratio and thus increasing the acidity character of Al-MCM-41 on the energy input of the process. To the best of our knowledge, no studies in the literature have evaluated the influence of Si/metal molar ratios on mesoporous acid catalysts using TGA under a H_2_ atmosphere. A few authors evaluated the efficiency of Al-MCM-41 catalysts for HDPE degradation [44,45] and reported a reduction in the degradation temperature range when these acidic mesoporous systems were present, but under inert atmosphere.

With regard to H-ZSM-5 data, a decrease in the Si/Al molar ratio from 500 to 11.5 leads to a diminishment of the energy required to degrade the HDPE. In fact, for the Si/Al ratios of 500, 40, and 11.5, the corresponding T_5%_ is reduced by 34, 95, and 115 °C, respectively. On the other hand, the characterization data show that a decrease in the Si/Al ratio results in a higher number of BASs and total acid sites. This indicates that, as within the Al-MCM-41 series, the ability to reduce the temperature required to convert HDPE shows an inverse dependence of the Si/Al ratio. Similar results have been described by Coelho et al. [46]. The authors investigated a series of ZSM-5 materials with different sodium contents (0, 50, 63, and 80%, where 0% corresponds to the acidic form) for the catalytic cracking of HDPE and found that increasing sodium contents (a lower concentration of acid sites), led to progressively higher degradation temperatures of the polymer. In fact, the degradation of HDPE with the proton form of ZSM-5 occurred at 80 °C lower than with ZSM-5 with 80% Na. Neves et al. [47] studied the effect of HY and NaY acidity on the catalytic cracking of PE by TGA and came to similar conclusions. Reducing the acidity of HY by adding Na ions to its structure leads to higher onset degradation temperatures of HDPE. In contrast, the introduction of H^+^ into NaY, leads to an easier degradation of the polymer. In both cases, there were no significant changes in the textural properties of the materials by introducing Na^+^ or H^+^ into the zeolite structures. Other authors confirm the positive effect of the catalyst acidity on the degradation process [48,49,50].

A distinct pattern is observed for the H-USY series. According to Table 2, the T_5%_ for H-USY zeolites with Si/Al = 2.9, 15, 30, and 40 decreases by 143, 162, 184, and 163 °C, respectively, when compared to the thermal run. In this case, the ability to reduce the temperature required to convert HDPE does not increase as the Si/Al ratio decreases. This contradicts the data for the H-ZSM-5 and Al-MCM-41 series and suggests that additional factors may influence HDPE degradation. Previous studies [16,26,27] have shown that the accessibility of the catalyst can play a crucial role in the HDC reaction, since the diffusion of the long HDPE chains inside of a microporous structure is slow and the reaction starts at the accessible active sites located on the outer surface of the zeolites [29,30]. In addition, as mentioned above, the H-USY zeolites with higher Si/Al ratios are obtained by successive dealumination procedures, which induces the formation of a secondary mesoporosity. The data in Table 1 show that within the H-USY series, increasing the Si/Al ratio leads to a decrease in both BASs and total acidity, but simultaneously to an increase in S_ext_, V_meso_, and V_meso_. Increased accessibility can reduce diffusion limiting pathways and may counterbalance the negative effect of a lower acidity. It is therefore expected that the performance of the catalysts in this series results from the balance between these two factors. The H-USY (30) catalyst, which has intermediate values for both acidity and porosity, was found to initiate the degradation of HDPE at the lowest temperature (240 °C), leading to the lowest energy requirement. Close T5% values (approximately 270 °C) are observed for H-USY (15) and H-USY (40), while H-USY (2.9) with the highest number of BASs but with the lowest S_ext_, V_meso_, and V_meso_ values shows the highest T5% (290 °C) in the series.

Overall, the TGA experiments for the Al-containing catalytic materials revealed two main trends. On the one hand, material series, where the structural and textural properties do not change significantly with the Si/Al ratio, i.e., Al-MCM-41, and H-ZSM-5, show a direct relationship between the degradation temperature range and the Si/metal ratio. In this case, the most promising systems with the lowest energy consumption correspond to the lowest Si/Al ratio and a high number of BASs. On the other hand, for the material series where the textural properties of the catalysts vary with the Si/Al ratio, i.e., the H-USY series, the performance of the catalysts results from the balance between the acidic and textural properties and no direct relationship between the Si/Al ratio and the degradation temperature range for HDPE is observed.

### 2.3. Hydrocracking Experiments

**Figure 2:**
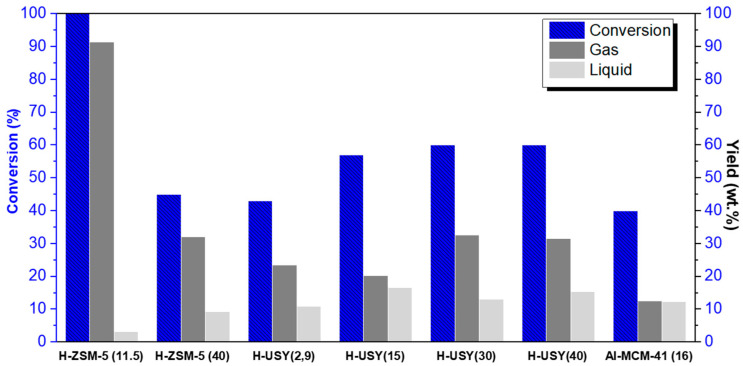
Yields in gas and liquid products obtained from the hydrocracking of HDPE over H-ZSM5 and H-USY with distinct Si/Al ratios and Al-MCM-41 (16) (T = 300 °C, t = 60 min, P_H2i_ = 20 bar, and HDPE/catalyst mass ratio= 8/2).

Based on TGA data, the most promising catalytic systems for HDPE degradation in terms of energy consumption were identified. These include (from most to least favorable) H-USY (30), H-USY (40), H-USY (15), H-USY (2.9), Al-MCM-41(16), H-ZSM-5 (11.5), and H-ZSM-5 (40). In order to gain a deeper insight into the catalytic behavior and to obtain data on conversion, product yields, and product distribution, these catalysts were evaluated in a batch autoclave reactor. The HDC experiments were conducted at a temperature of 300 °C, a pressure of 20 bar, and a duration of 60 min, with a polymer to catalyst ratio of 8:2. Figure 2 illustrates the conversions and yields in gas and liquid products of the selected catalysts.

Regarding the HZSM-5 zeolites, it can be seen that at Al/Si = 11.5 the full conversion of the HDPE is achieved, while at Si/Al = 40 the conversion decreases to 45%. These results confirm the positive effect of decreasing Si/Al ratio and increasing acidity on the H-ZSM-5 series, as observed in the preliminary TGA studies. In addition, the Si/Al ratio also affects the yields in gaseous and liquid products. The H-ZSM-5 (11.5) zeolite shows a high tendency to produce gaseous products (≥90 wt.%), while for H-ZSM-5 (40) the gaseous fraction decreases to 35 wt. %. This behavior is probably due to the lower number of acid sites (BASs) on H-ZSM-5 (40), which leads to a decrease in the HDC reaction rate [51]. Overall, for H-ZSM-5 zeolites, both the conversion and the yields of the gaseous and liquid products are affected by the Si/Al ratio, with the hydrocracking ability being directly correlated with the acidity and inversely dependent on the Si/Al ratio.

Consistent with the preliminary TGA evaluation of the catalytic potential within the H-USY series, no direct relationship between the Si/Al ratio and the catalytic activity was observed. In fact, the less efficient catalyst (with the lowest conversion, 42%), is the one with an Al/Si ratio of 2.9 while at higher Si/Al ratios, the conversion increases up to approximately 60%. In terms of products obtained, both H-USY 30 and 40 show the highest gas yield (~30 wt. %) as a result of their higher cracking ability. As stated above, along the H-USY series, the accessibility of the catalytic acid sites varies with the Si/Al ratio. Accordingly, the catalytic performance of these materials results from the balance between the acidic and textural properties and no direct correlation between the Si/Al ratio and HDPE conversion/degradation temperature for HDPE is observed. Indeed, if improved textural properties, higher S_ex_, V_meso_, and Φ_meso_, resulting in an increased accessibility of bulky polymer molecules to the internal active sites, can overcome a low density and strength of the acid sites, higher catalytic performance can be achieved at intermediate Si/Al values. It is also important to note that the formation of coke precursors, and consequently the deactivation of the catalyst during the reaction, is another important factor that can strongly impact the catalytic performance, as will be discussed later.

To the best of our knowledge, there are no studies in the literature which have evaluated the influence of the Si/Al ratio in H-USY zeolites for the hydrocracking of plastic feedstocks. Nevertheless, Cardona et al. [52] investigated the catalytic cracking of polypropylene over H-USY zeolites with distinct Si/Al ratios in a semi-batch reactor under inert conditions. In agreement with our results, they concluded that neither the total amount nor the strength of the acid sites are unique factors affecting the reaction and suggested that the increase in accessibility in H-USY zeolites, resulting from a secondary mesoporosity, improves the catalytic activity.

With respect to Al-MCM-41 (16), a conversion of 40%, and a yield in gaseous and liquid products of 12 wt.% were obtained. The observed conversion is comparable to the values obtained for the H-USY (2.9) and H-ZSM-5 (40) zeolites, i.e., 42 and 44%, respectively, but still far from the best catalytic performances. Due to its mesoporous nature, Al-MCM-41(16) has a better accessibility than H-USY and HZM-5 zeolites. Therefore, the lower catalytic performance cannot be explained by diffusion limitations. However, Aguado et al. found that mesoporous silicas have weaker acid properties compared to zeolites, even after Al incorporation ion [53]. The acidity data in this study also reveals the same pattern. Indeed, the number of BASs is lower than for the H-USY series and much lower than for the H-ZSM-5 series. Moreover, while Al-MCM-41 exhibits 7% strong BASs, the best H-USY and HZM-5 zeolites (H-USY (30), H-USY (40), and HZM-5 (11,5)) show at least 36% strong BASs. Therefore, it is likely that the low number of strong BASs might explain the lower hydrocracking ability. A high content of strong acid sites has been previously associated with a high performance for the catalytic cracking of plastic waste [54]. As will be discussed later, the high tendency to form coke deposits within large pore materials, which can lead to a rapid deactivation of this catalytic system, is another important factor to consider in the overall catalytic performance.

To the best of our knowledge, only Munir et al. [55] applied a mesostructured acidic material (Al-SBA-16) as a catalyst for the hydrocracking of municipal solid waste and compared its catalytic performance with the USY zeolite. The results showed that Al-SBA-16 converts 25% of plastic feedstock at 375 °C, while H-USY allows a higher conversion (~35%).

**Figure 3:**
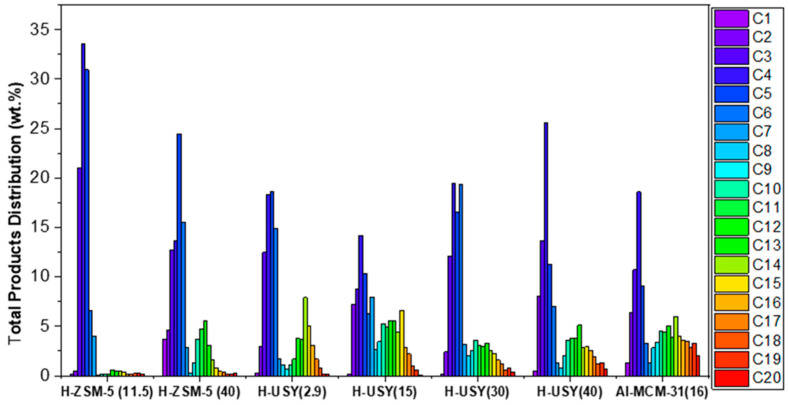
Total products distribution of HDPE hydrocracking over H-ZSM5 and H-USY with distinct Si/Al molar ratios and Al-MCM-41 (16) (T = 300 °C, t = 60min, PH2i = 20 bar, and HDPE/catalyst mass ratio = 8/2).

**Figure 4:**
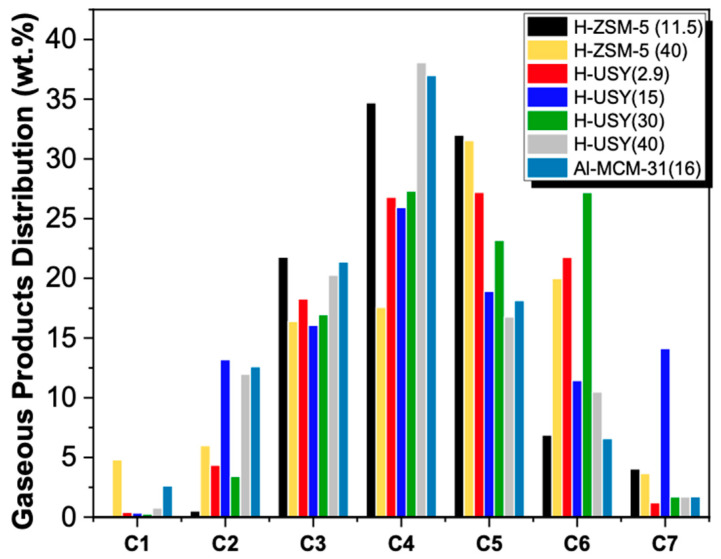
Gaseous products distribution of HDPE hydrocracking over H-ZSM-5 and H-USY with distinct Si/Al ratios and Al-MCM-41 (16) (T = 300 °C, t = 60 min, PH2i = 20 bar, and HDPE/catalyst mass ratio = 8/2).

**Figure 5:**
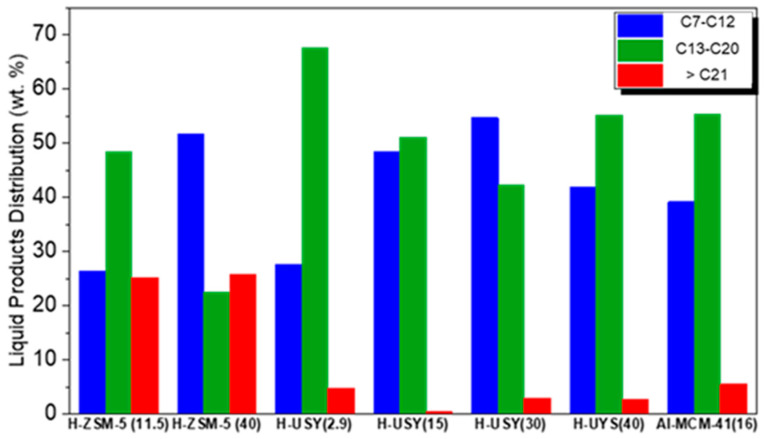
Liquid products distribution of HDPE hydrocracking over H-ZSM-5 and H-USY with distinct Si/Al ratios and Al-MCM-41 (16) (T = 300 °C, t = 60 min, PH2i = 20 bar, HDPE/catalyst mass ratio = 8/2).

The total products distribution obtained over Al-MCM-41(16), and H-ZSM-5 and H-USY with different Si/Al ratios, are shown in Figure 3. The detailed compositions of the gaseous and liquid fractions are displayed in Figure 4 and Figure 5, respectively.

According to Figure 3, H-ZSM-5 (11.5) shows a product distribution composed predominantly of light hydrocarbons in the C_3_-C_5_ range. This distribution results from the high number of strong acid sites and the shape selectivity exerted by its porous network, which favors an end-chain cracking mechanism. In fact, according to the literature, strong acid sites catalyze the degradation of heavier hydrocarbons into lighter gaseous products to a greater extent than weak ones [56] and smaller pore sizes also tend to favor the production of lighter hydrocarbon molecules [16]. Increasing the Si/Al molar ratio of H-ZSM-5 leads to a broader distribution of the products, with hydrocarbons from C_1_ to C_17_, and with two maxima at C_5_ and C_12_. The presence of heavier hydrocarbon fractions may be related to the much lower acidic character of H-ZSM-5 (40) compared to that of H-ZSM-5 (11.5), in terms of total acid sites (245 vs. 760 μmol Py/g), BASs (215 vs. 649 μmol/g), and strong BASs (19% vs. 59%). With respect to the gaseous fraction (Figure 4), a slight shift towards hydrocarbons with a high number of carbon atoms is observed. However, it should be noted that for H-ZSM-5 (40), a high amount of C_1_ and C_2_ is observed in the gaseous fractions (>9 wt. %). Coelho et al. [46] also evaluated the influence of the acidity of H-ZSM-5 on the gaseous products obtained in the catalytic cracking of HDPE. Their results showed a similar trend, with an increase in zeolite acidity (decrease in the Si/Al molar ratio) leading to an increase in the C_3_–C_5_ fraction. The authors also reported higher amounts of CH_4_ for the least acidic H-ZSM-5 zeolite. Regarding the composition of the liquid products, shown in Figure 5, increasing the Si/Al molar ratio results in a high selectivity towards the gasoline range.

The H-USY series shows a broader product distribution than the H-ZSM-5 series, with hydrocarbons from C_1_ to C_20_, and with a significant contribution from hydrocarbons in the C_7_–C_20_ range (Figure 3). This is an expected result since H-USY catalysts have larger pores than H-ZSM-5 materials. According to the literature [56,57], the pore size of a catalyst plays a crucial role in the products distribution in catalytic reactions, particularly in processes such as hydrocracking and/or catalytic cracking. Catalysts with smaller pores tend to favor reactions that produce lighter hydrocarbon products, such as gases and light liquids, while the larger pore size allows a wider range of molecules to react, often resulting in a more diverse product distribution, including both lighter and heavier hydrocarbons.

The gaseous products (Figure 4) are composed of C_2_ to C_7_ hydrocarbons, with C_4_ as the predominant component. In agreement with other studies, a relatively small amount of C_1_ (<2.5 wt.%) is obtained [13,58]. The liquid products are mainly composed of hydrocarbons with boiling points between 98 and 344 °C (C_7_ to C_20_) and minor amounts of heavier hydrocarbons (≥C_21_) (Figure 5). Among the H-USY series, the H-USY (2.9) exhibits the highest selectivity towards the diesel range fraction (~70 wt.%) and a product distribution shifted towards heavier fractions, as a result of its low activity for the HDC reaction. Munir et al. [55], investigated the product distribution obtained in the hydrocracking of municipal solid waste promoted by USY (Si/Al = 15) and also reported a higher selectivity for the diesel fraction. In turn, Ochoa et al. [8] studied the effect of the acidity of silica-alumina catalysts on the HDC of medium density polyethylene, by varying the Si/Al ratio (25% SiO_2_:Al_2_O_3_; 50% SiO_2_:Al_2_O_3_, and 75% SiO_2_:Al_2_O_3_, where percentages are related to Al content). The results show that 25% SiO_2_:Al_2_O_3_ leads to a larger hydrocarbon range C_5_–C_22_ (~70 wt.%) than the non-acidic silica, but an increase in the aluminum content to 50 or 75% shifts the products to lighter hydrocarbons (C_5_–C_12_).

With respect to the Al-MCM-41 (16) catalyst, a product distribution similar to H-USY (40) is obtained. The main component of the gaseous products is the C_4_ hydrocarbon fraction, while the liquid products show a high selectivity towards the diesel range (55 wt.%). This mesoporous silica and the H-USY (2.9) zeolite exhibit the highest amount of liquid hydrocarbons with a carbon atom number above 20 (~6 wt.%).

**Figure 6:**
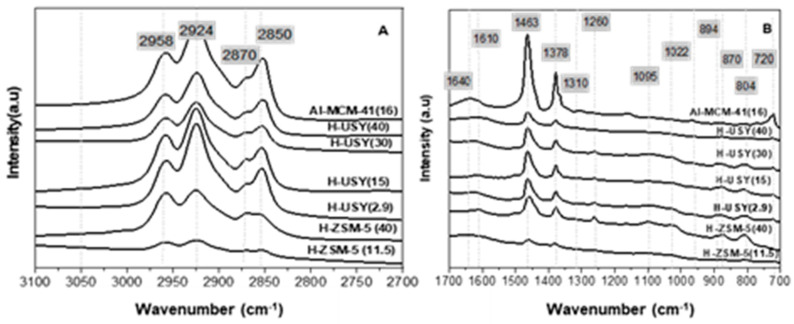
FTIR spectra (**A**) from 2700–3100 cm^−1^ and (**B**) from 700–1700 cm^−1^ of liquid product obtained from HDPE hydrocracking over H-ZSM5 and H-USY with distinct Si/Al molar ratios and Al-MCM-41 (16) (T = 300 °C, t = 60 min, PH2i = 20 bar, and HDPE/catalyst mass ratio = 8/2).

The presence of different families of compounds in the liquid fraction was assessed via FTIR. The spectra of the liquid products are displayed in Figure 6.

The typical vibrational bands of asymmetric and symmetric methyl (2958 and 2870 cm^−1^) and methylene (2924 and 2855 cm^−1^) groups, characteristic of alkanes, are clearly observed [59,60]. The presence of these compounds is also identified by the bands at 1463 and 1368 cm^−1^, which are characteristic of the asymmetric and symmetric deformation stretching of -CH_3_ groups. Instead, the absence of bands between 3040–3070 cm^−1^ and between 1640–1650 cm^−1^, characteristic of alkene CH stretching and C = C bond stretching [59], suggests the absence of olefins in the products, as typically observed in HDC reactions [23]. A negligible amount of olefinic compounds was also observed by other authors [27,61,62], who studied the hydrocracking of waste plastics over a monofunctional acidic catalyst. This is a major advantage of catalytic hydrocracking compared to catalytic cracking carried out under a N_2_ atmosphere, which produces hydrocarbon streams with high olefinic content.

It is well known that heavy hydrogen-deficient molecules can be formed during HDC reactions [63,64]. These carbonaceous products are responsible for poisoning or blocking the access to the active sites leading to catalyst deactivation and severely affecting the catalytic performance [65]. The rate of coke formation depends on several factors, namely the reaction medium, operating conditions, and feedstock properties. In addition, the properties of the catalyst, namely its structure and the number and density of the acid sites also have a determinant role in the production of these carbonaceous deposits [66,67].

**Figure 7:**
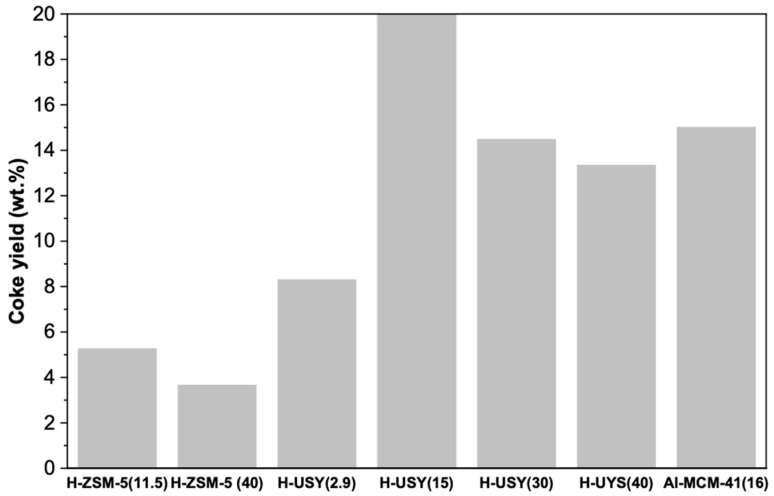
Yield in coke of HDPE hydrocracking over H-ZSM5 and H-USY with distinct Si/Al ratios and Al-MCM-41 (16) (T = 300 °C, t = 60 min, PH_2i_ = 20 bar, and HDPE/catalyst mass ratio = 8/2).

To further examine the impact of the catalyst’s physical and chemical properties on the rate of coke formation and its subsequent effect on the catalytic performance, the yields of coke obtained in the hydrocracking of HDPE over H-ZSM-5 and H-USY with varying Si/Al ratios and Al-MCM-41(16) are presented in Figure 7.

The data obtained indicate that H-USY zeolites (FAU structure) produce significantly higher amounts of coke than H-ZSM-5 ones (MFI structure). Indeed, the H-USY (15) zeolite exhibits the highest yield in coke, followed by H-USY (30), H-USY (40), H-USY (2.9), and only then by the H-ZSM-5 (11.5) and H-ZSM-5 (40) zeolites. The markedly reduced quantities of carbonaceous deposits formed on H-ZSM-5 structures corroborate the critical role of the porous structure in limiting coke formation, as previously discussed in reference [16]. In fact, the occurrence of secondary bimolecular reactions is reduced within the medium-size pore HZSM-5 zeolite, which limits the formation of bulky reaction intermediates that lead to coke. In contrast, the presence of supercages in the large-pore H-USY zeolites, while facilitating the access of polymer chains to the active sites, results in a faster rate of coke formation and subsequent catalyst deactivation. Pore size is therefore critical to optimizing catalyst performance; the pores must be large enough to facilitate the reaction, yet narrow enough to limit secondary reactions.

Furthermore, an increase in the Si/Al molar ratio within the H-ZSM-5 series is accompanied by a reduction in the number of total acid sites and BASs, resulting in a slight decrease in coke yield from 5.3 to 3.7 wt.%. This result is in accordance with expectations, since the greater the number and strength of acidic sites, especially BASs, the greater the rate of coke formation [68]. On one hand, a higher density of Brønsted acid sites (BASs) results in an increased number of successive reactions along the diffusion pathway, thereby promoting more condensation reactions and accelerating coke formation. On the other hand, the stronger the centers the faster the reaction steps and the rate of formation of the coke precursors.

A comparable trend is observed in H-USY zeolites with Si/Al molar ratios of 15, 30, and 40. Indeed, the zeolite with the highest density and strength of protonic acid sites [H-USY (15)], exhibits the highest coke yield (~20 wt.%), whereas the one with the lowest density and strength of acid sites [H-USY (40)] leads to a lower production of carbonaceous deposits (~13 wt.%). An exception to this pattern is observed with H-USY (2.9), which, despite having the second highest BAS concentration and a substantial proportion of strong BASs (79%), leads to the lowest production of hydrogen-deficient hydrocarbons (~8 wt.%). A possible explanation for this is the aforementioned lower accessibility of this zeolite compared to the other zeolites in the H-USY series.

The Al-MCM-41(16) zeolite exhibits a coke yield of 15 wt.%, which is comparable to the values observed for H-USY (30) and (40). Despite its weaker acidity compared to H-USY and H-ZSM-5 structures (especially in terms of BASs and strong BASs), its mesoporous structure, with accessible acid sites, facilitates the formation and growth of coke molecules. These results are in agreement with those previously reported by Aguado et al. [44]. The authors studied the catalytic cracking of LDPE, HDPE, and recycled PE over H-ZSM-5 and mesostructured acidic materials (Al-MCM-41 and Al-SBA-15). The deactivation effect was found to be much more pronounced in materials with larger pores but weaker acid properties (Al-MCM-41 and Al-SBA-15) than in materials with a microporous structure but strong acidity (H-ZSM-5). This suggests that accessibility plays a dominant role in coke formation.

Finally, it is important to emphasize that, in addition to the textural and acidic characteristics of the catalyst, deactivation by coke formation is another critical factor in the overall catalytic performance, as it may prevent the full potential of a given catalyst from being achieved. Indeed, despite the highest potential demonstrated by H-USY (15) in the preliminary TGA studies (highest decrease in degradation temperature range), the reactor batch HDC tests showed a much lower performance (60% conversion compared to full conversion for HZSM-5 (11.5)). Most likely, deactivation caused by a much higher coke yield in the former case (20%) vs. the latter (5%) and an expected, more pronounced deactivation for the longer reaction times in the batch autoclave reactor contribute to this fact.

## 3. Materials and Methods

### 3.1. Materials

The commercial high-density polyethylene (HDPE, M_W_ = 155,000 g/mol; MWD = 5.4; d = 0.95 g/cm^3^, and T_m_ = 140 °C) used in this study was supplied by Repsol in a powder form, free of any additives.

The H-USY zeolites, with Si/Al molar ratios of 2.9, 15, 30, and 40) and the NH_4_-ZSM-5 zeolites, with Si/Al molar ratios of 11.5, 40, and 500) were provided by Zeolyst (Conshohocken, PA, USA).

The gallium nitrate [Ga (NO_3_)_3_] and ammonium nitrate [NH_4_NO_3_] used for the synthesis of Al- and Ga-modified MCM-41 catalysts were purchased from Sigma Aldrich (St. Louis, MO, USA) and Merck (Darmstadt, Germany), respectively, both with a purity of 99%.

The n-hexane (*n*-C_6_) used as a solvent for the recovery of the liquid products was supplied by VWR Chemicals with a purity of 99.8%.

### 3.2. Catalyst Preparation

The zeolite in ammonia (NH_4_) form was calcined to the corresponding proton form (H) according to the method reported in a previous work [27].

The pure siliceous MCM-41 was prepared according to the method described by Kim et al. [69]. In order to enhance the surface acidity, MCM-41 was modified with aluminum (Al) and gallium (Ga).

The Al-MCM-41 materials were prepared by direct synthesis as previously described by Lindlar et al. [70]. Samples with different Si/Al ratios were synthetized by adjusting the aluminum content in the synthesis gel. The template was partially removed by extraction with a solution of 0.1 M NH_4_NO_3_ in 96% ethanol, at reflux temperature for 2 h. After drying, the product was calcined under a flux of dry air at 550 °C for 10 h. The temperature was increased from 25 to 550 °C, at a rate of 1 °C/min.

The Ga-MCM-41 materials were synthetized by adapting the method used for Al-MCM-41 synthesis and using Ga (NO_3_)_3_ as the metal source. The template was partially removed by extraction with a 0.1 M NH_4_NO_3_ solution in 96% ethanol at reflux temperature for 2 h. After drying, the products were calcined under a flow of dry air at 550 °C for 12 h [71]. The Al-MCM-41 supports have Si/Al ratios of 16 and 30, while the Ga-modified materials have Si/Ga ratios of 63 and 82.

For all materials the Si/metal ratio is given in parentheses after the catalyst name.

### 3.3. Catalyst Characterization

The elemental analysis of the catalysts was performed by Inductively Coupled Plasma—Optical Emission Spectroscopy (ICP-OES), using a HORIBA Jobin Yvon- ACTIVA M spectrometer (HORIBA Scientific, France). The operating conditions used for the quantitative determination of Si, Al, and Ga contents were as follows: 1000 W RF power, 12 L/min plasma flow, 0.2 L/min auxiliary flow, 0.02 L/min nebulizer flow, and 1.0 mL/min sample uptake rate.

The structure of the zeolites and mesostructured acidic materials was evaluated by Powder X-Ray diffraction analysis (PXRD). The diffraction patterns were obtained in a Bruker AXS Advance D8 (Billerica, MA, USA) diffractometer equipped with a 1D detector (SSD 160) and using a Ni filter, with a CuKα radiation source (λ = 1.5406 nm) and operating at 40 kV and 30 mA. The scanning range was defined from 5° to 80° for the H-USY and H-ZSM-5 zeolites and from 1 to 5° (2 theta) for the MCM-41 based materials, with a step size of 0.03°/2 s.

The textural properties of the catalysts were determined by nitrogen (N_2_) sorption measurements at -196 °C using an Autosorb IQ apparatus from Quantachrome (Boynton Beach, FL, USA). Prior to the measurements, the materials were degassed under vacuum at 90 °C for 1 h and then heated at 350 °C for 5 h. The external surface area (S_ext_) and the micropore volume (V_micro_) were calculated using the t-plot method, whereas the total pore volume was determined from the adsorbed volume of nitrogen at a relative pressure (P/P_0_) of 0.95. The mesoporous volume (V_meso_) was obtained as the difference between V_total_ and V_micro_. The average of the pore diameter was determined from the desorption data using the Barrett-Joyner-Halenda (BJH) method.

The quantification of the density, strength, and nature of the acid sites was performed by FT-IR spectroscopy using pyridine as a probe molecule. Self-supported wafers were placed in an IR quartz cell and evacuated under secondary vacuum (10^−6^ Torr) at 300 °C for 2 h prior to pyridine adsorption at 150 °C (equilibrium pressure ~1.5 Torr). The subsequent desorption of pyridine was performed under secondary vacuum at 150 and 350 °C, for 30 min. The FTIR spectra were recorded on a ThermoNicolet Nexus 670 (Thermo Electron Corporation, Waltham, MA, USA) instrument (64 scans, 4 cm^−1^ resolution). The background spectrum, recorded under identical operating conditions, was automatically subtracted from each sample spectrum. The acid sites were classified according to their nature as Brønsted and Lewis acid sites, BASs and LASs, respectively. The concentration of BASs was determined from the FT-IR adsorption band at 1545 cm^−1^, typical of pyridinium ions (PyH^+^), while the concentration of LASs was calculated from the FT-IR adsorption band at 1454 cm^−1^, characteristic of coordinatively adsorbed pyridine [72]. For these quantitative measurements, pyridine extinction molar coefficients from Emeis [73] were used.

### 3.4. Catalytic Performance Evaluation

#### 3.4.1. Sample Preparation

The samples used for the preliminary degradation and HDC experiments were prepared by compression molding. First, the HDPE and the catalyst, both in powder form, were mechanically mixed in a polymer to catalyst mass ratio of 8 to 2, and then heated in a press at 140 °C and 3 tons of pressure for 5 min [26].

#### 3.4.2. Preliminary Degradation Experiments

The preliminary degradation experiments were performed in a Setaram 92–16.18 simultaneous TGA-DSC apparatus. For all of the experiments, approximately 10 mg of sample (HDPE or HDPE + catalyst) was heated from room temperature to 700 °C at a heating rate of 10 °C/min. The experiments were performed with an H_2_ flow rate of 30 mL⋅min^−1^. To avoid the presence of oxygen (O_2_), a nitrogen (N_2_) purge was performed before each experiment. The mass loss data was calculated based on the total mass, i.e., the mass of the polymer and catalyst. In this case, the residual mass corresponds to the catalyst and any coke deposits.

#### 3.4.3. Hydrocracking Experiments

The HDC tests were performed in a 100 mL batch autoclave reactor from Autoclave Engineering equipped with a magnetic stirrer, as described elsewhere [16]. The reactor was filled with 1 g of the sample prepared by compression molding and flushed three times with N_2_ at 20 bar to ensure the absence of oxygen. The reactor was then pressurized with 20 bar of H_2_ at room temperature and heated to 300 °C at a heating rate of 1.5° C/min. The final temperature was maintained for 60 min. The gaseous products formed during the reaction were sampled in a glass ampoule of known volume, after venting the reactor to atmospheric pressure. Cis-2-butene was used as the internal gas by systematically adding a fixed amount of this gas to the ampoule. The remaining products were removed from the reactor and the liquid fraction was separated from the solid one by a Soxhlet type extraction (P_atm_, T = 69 °C), using n-hexane (n-C_6_H_14_) as a solvent (V = 40 mL). The solid fraction was then dried overnight at 60 °C to evaporate the n-C_6_H_14_ solvent. The yields for products and the process conversion were determined using the following equations:

(1) $$Conversion\left(\%\right)=\frac{m_{HDPE}-m_{unconverted\;HDPE}}{m_{HDPE}}\times 100$$

(2) $$Gas\;yield\left(wt.\%\right)=\frac{m_{Gas}}{m_{HDPE}}\times 100$$

(3) $$Liquid\;yield\left(wt.\%\right)=\frac{m_{Hexane\;soluble}}{m_{HDPE}}\times 100$$

(4) $$Coke\;yield\left(wt.\%\right)=\frac{m_{Coke}}{m_{HDPE}}\times 100$$

#### 3.4.4. Products Characterization

The composition of the gaseous fraction was analyzed by gas chromatography (GC) equipped with a flame ionization detector (FID) using a GC1000 DPC chromatograph and a capillary HP-PONA column (50 m × 0.2 mm × 0.5 um) from Agilent. The liquid products were characterized by a simulated distillation analyzer using a Hewlett Packard 5890 gas chromatograph equipped with an FID detector and a DB-2887 capillary column (10 × 0.53 mm × 0.3 um) from Agilent.

The solid fraction resulting from the HDC reaction was analyzed through two TGA cycles using a Setaram TGA-92 apparatus, in order to quantify the amount of unconverted HDPE as well as the carbon deposit over the catalyst. First, the solid fraction, containing unreacted plastic, coke, and the catalyst, was heated to 500 °C at a rate of 10 °C/min and held at this temperature for 1 h under nitrogen flow (30 mL/min), to remove the unreacted HDPE from the remaining solid components. The sample resulting from this first TGA cycle, consisting of coke and catalyst, was then heated to 800 °C at the same rate and held for 30 min under an air atmosphere (30 mL/min) to burn off all of the coke deposited on the catalyst. The amount of unreacted HDPE was determined by the difference between the initial and final sample masses from the first TGA cycle, while the amount of coke was calculated from the mass difference in the second TGA cycle.

## 4. Conclusions

In this work, the effect of microporous H-USY and H-ZSM-5 zeolites and mesoporous MCM-41 modified with Ga and Al, with distinct Si/Metal ratios and consequently different, structural, textural, and acid properties, were evaluated as catalytic systems for HDPE hydrocracking.

The characterization data indicate that MCM-41 modified with Ga and Al shows promising structural and textural properties, in terms of an enhanced accessibility to the polymer macromolecules and reduced diffusional limitations during the reaction, when compared to microporous zeolites. Nevertheless, the acid properties of these materials are strongly dependent on the metal added to the structure, Ga or Al. The introduction of Ga promotes the formation of Lewis acid sites (LASs), while the presence of Al favored the Brønsted ones (BASs), which were found to be essential for the HDC reaction. The number of BASs increases with the decrease in the Si/Al ratio. For the H-ZSM-5 series a similar trend is observed, i.e., increasing the Si/Al molar ratio leads to a reduction of LASs, BASs, and strong BASs, while the textural properties do not change significantly. A distinct behavior is observed for the H-USY series. In this case, an increase in the Si/Al molar ratio also leads to a decrease in the density of the acid sites (BASs and LASs), but the accessibility to the active sites is improved (higher Sext and Vmeso), due to the dealumination procedure used in their synthesis method.

A subsequent evaluation of the catalytic performance of the different materials by TGA studies and HDC catalytic tests in an autoclave reactor clearly demonstrated the fundamental role of the catalyst’s acidity (especially the number of BASs and strong BASs) and active sites’ accessibility in the hydrocracking reaction of HDPE.

Two distinct situations were identified for the Al-containing material series with catalytic properties. On the one hand, for the Al-MCM-41 and H-ZSM-5 series (whose structural properties do not vary significantly with the Si/Al ratio) a higher HDPE conversion/lower degradation temperature is obtained at the lowest Si/Al ratio, i.e., a high performance for the HDC of HDPE is associated with a high concentration of BASs and strong BASs. On the other hand, the catalytic performance for the H-USY series, which shows a change in both acidity and accessibility with the Si/Al ratio, results from the balance between the acidic and textural properties and no direct correlation between the Si/Al ratio and HDPE conversion/degradation temperature for HDPE is observed.

The distribution of products was found to vary with the catalyst type and the Si/Al ratio. It is worth noting that H-ZSM-5 (11.5), which shows the highest hydrocracking activity in the HDC tests in the batch autoclave reactor, giving rise to full HDPE conversion, shows a product distribution composed predominantly of light gas hydrocarbons in the C_3_–C_5_ range. On the other hand, catalysts with lower cracking ability (conversions in the range 40–60%) show a much broader distribution of the products, with hydrocarbons from C1 to C20, and with a significant contribution from liquid products in the C_7_–C_20_ range.

It is also important to emphasize that, in addition to the textural and acidic properties of the catalyst, the rate of coke formation is another determinant factor to ensure a comprehensive analysis of the catalytic performance, as catalyst deactivation by coke formation may prevent the full potential of a given catalyst from being realized.

The findings of this study underscore the importance of catalyst design in the hydrocracking of HDPE, particularly concerning the balance between acidity and accessibility. The study provides valuable insights into the development of efficient catalytic systems for plastic waste management, with potential implications for both environmental sustainability and the chemical industry.

## Figures and Tables

**Table 1:** Textural, structural, and acidic properties of Al- and Ga-modified MCM-41, H-USY, and H-ZSM-5 with distinct Si/metal ratios obtained from N_2_ sorption and PXR measurements.

Catalyst	S_BET_ (m^2^/g)	S_ext_ (m^2^/g)	V_micro_ (cm^3^/g)	V_meso_ (cm^3^/g)	V_total_ (cm^3^/g)	Φ _meso_ (nm)	PyL (μmol/g)		PyH^+^ (μmol/g)		Total Acidity (μmol/g)
							150 °C	350 °C	150 °C	350 °C	
Si-MCM-41	1007	-	0.0	0.85	0.85	3.4	-	-	-	-	-
Al-MCM-41 (16)	1092	-	0.0	0.82	0.82	3.3	232	159	113	8	345
Al-MCM-41 (30)	1047	-	0.0	0.87	0.87	3.0	162	117	86	33	248
Ga-MCM-41 (63)	734	-	0.0	0.63	0.63	3.4	13	3	0	0	13
Ga-MCM-41 (82)	1016	-	0.0	0.69	0.69	3.4	31	19	0	0	31
H-USY (2.9)	-	87	0.19	0.14	0.33	-	200	87	194	134	394
H-USY (15)	-	189	0.25	0.23	0.48	-	83	60	230	103	313
H-USY (30)	-	193	0.21	0.25	0.45	-	30	27	156	57	186
H-USY (40)	-	251	0.21	0.25	0.46	-	14	11	96	35	110
H-ZSM-5 (11.5)	-	114	0.13	0.10	0.23	-	111	89	649	384	760
H-ZSM-5 (40)	-	129	0.12	0.10	0.22	-	30	39	215	40	245
H-ZSM-5 (500)	-	75	0.11	0.07	0.18	-	n.d.	n.d.	n.d.	n.d.	n.d.

**Table 2:** Temperature at which mass loss is 5, 50, and 95% (T_5%_, T_50%_, and T_95%_) for HDPE degradation under H2 atmosphere over Al- and Ga-modified MCM-41 materials, H-ZSM-5, and H-USY zeolites with distinct Si/metal ratios.

Sample	T_5%_ (°C)	T_50%_ (°C)	T9_5%_ (°C)
HDPE	433	478	488
Si-MCM-41	431	474	468
Al-MCM-41 (16)	307	389	421
Al-MCM-41 (30)	420	455	471
Ga-MCM-41 (63)	429	466	480
Ga-MCM-41 (82)	428	470	488
H-USY(2.9)	290	378	406
H-USY(15)	271	370	410
H-USY(30)	249	340	382
H-USY(40)	270	357	399
H-ZSM-5 (11.5)	318	407	428
H-ZSM-5 (40)	338	405	422
H-ZSM-5 (500)	399	455	471

## Data Availability

Data are contained within the article or Supplementary Materials.

## Supplementary Materials

The following supporting information can be downloaded at: https://www.mdpi.com/article/10.3390/molecules29174248/s1, Figure S1: Nitrogen sorption isotherms (**A**) and pore size distribution (PSD) (**B**) of Al and Ga modified MCM-41 materials with distinct Si/metal ratios (Si/Al=16 and 30; Si/Ga=63 and 82); Figure S2: Nitrogen sorption isotherms of H-USY and H-ZSM-5 (A) with distinct Si/Al ratio; Figure S3: TGA profiles obtained for HDPE degradation under H2 atmosphere over Al and Ga modified MCM-41 materials (**A**,**B**), H-ZSM-5 (**C**,**D**) and H-USY (**E**,**F**) zeolites with distinct Si/metal ratios.
