# Supplementary material for: Catalyst Accessibility and Acidity in the Hydrocracking of HDPE: A Comparative Study of H-USY, H-ZSM-5, and MCM-41 Modified with Ga and Al
Source: Molecules. 2024 Sep 7;29(17):4248. doi: 10.3390/molecules29174248 (PMC11397643; doi:10.3390/molecules29174248)
Supplement: Supplementary file 1 [file molecules-29-04248-s001.zip › molecules-3167318-supplementary.pdf]

Supporting Information

Catalyst Accessibility and Acidity in the Hydrocracking of HDPE: A Comparative Study of H-USY, H-ZSM-5, and MCM-41 Modified with Ga and Al

Cátia S. Costa[1*], M. Rosário Ribeiro[1*], João M. Silva[1,2]

1 *Centro de Química Estrutural, Institute of Molecular Sciences, Departamento de Engenharia Química, Instituto Superior Técnico, Universidade de Lisboa, Portugal*

2 *Chemical Engineering Department, Instituto Superior de Engenharia de Lisboa, Instituto Politécnico de Lisboa, Portugal*

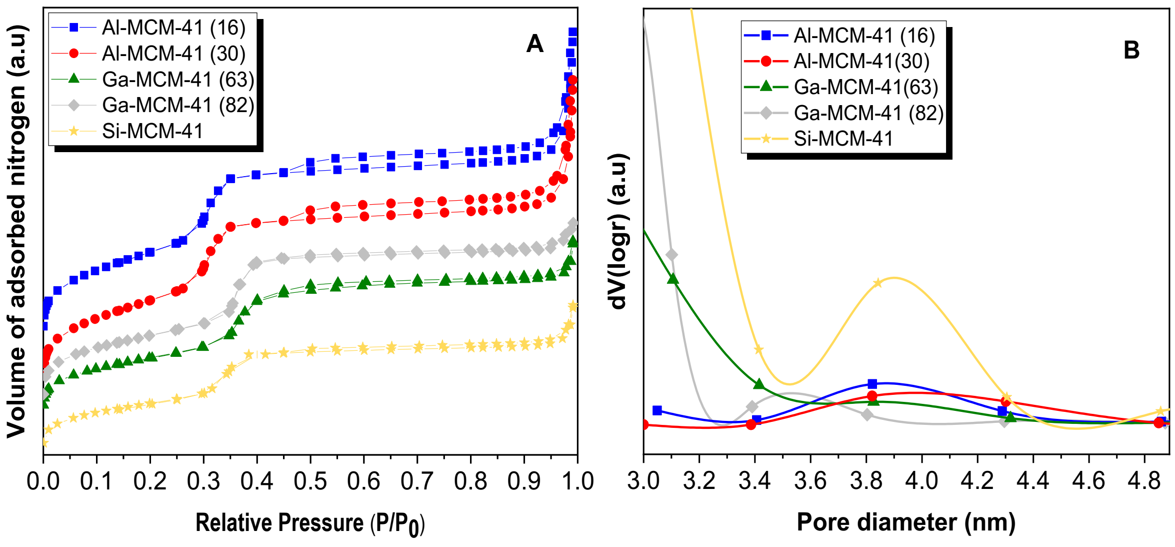

Figure S.1- Nitrogen sorption isotherms (A) and pore size distribution (PSD) (B) of Al and Ga modified MCM-41 materials with distinct Si/metal ratios (Si/Al=16 and 30; Si/Ga=63 and 82).

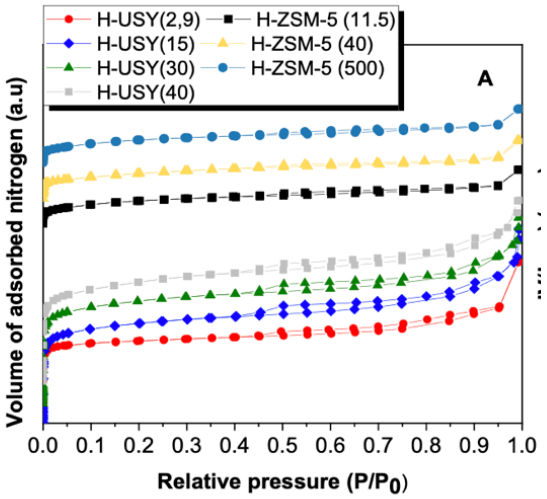

Figure S.2- Nitrogen sorption isotherms of H-USY and H-ZSM-5 (A) with distinct Si/Al ratio.

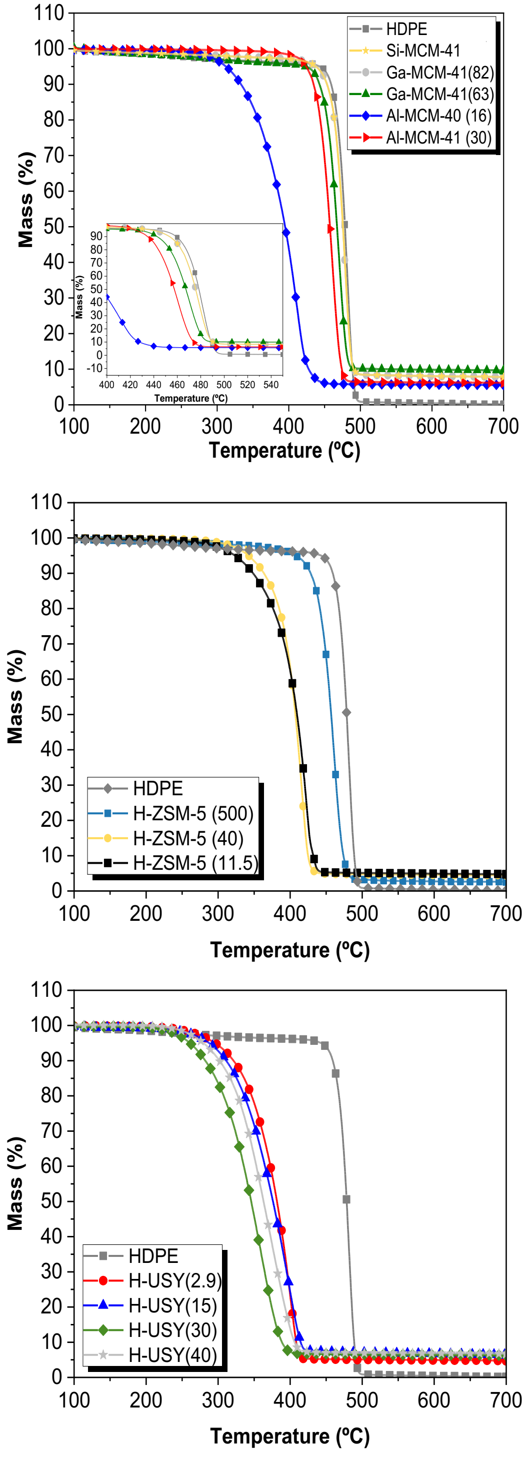

Figure S.3- TGA profiles obtained for HDPE degradation under H_2 atmosphere over Al and Ga modified MCM-41 materials (A, B), H-ZSM-5 (C,D) and H-USY (E,F) zeolites with distinct Si/metal ratios
